# Four-Year Nutritional Outcomes in Single-Anastomosis Duodeno-Ileal Bypass with Sleeve Gastrectomy Patients: an Australian Experience

**DOI:** 10.1007/s11695-023-06461-1

**Published:** 2023-01-26

**Authors:** Ravi Rao, Munish Mehta, Devesh Ramesh Sheth, Gabrielle Hogan

**Affiliations:** 1 Perth Surgical & Bariatrics, 30 Churchill Avenue, WA 6008 Subiaco, Perth, Australia; 2 Big Data Scientists, Perth, Australia; 3 Clinicare Compounding Pharmacy, Maylands, Australia; 4 The University of Western Australia, Perth, Australia

**Keywords:** SADI-S, Nutritional deficiencies, Bariatric, Supplement, Australia, Malnourishment

## Abstract

**Abstract:**

Nutritional deficiencies following malabsorptive surgeries are a major concern.

**Purpose:**

To present clinical-based, mid-term nutritional outcomes in single-anastomosis duodeno-ileal bypass with sleeve gastrectomy (SADI-S) patients using a nutritional supplement based on the American Society for Metabolic & Bariatric Surgery (ASMBS) guidelines.

**Setting:**

Single private institute, Australia.

**Materials and Methods:**

Data from 196 patients who underwent a primary SADI-S by a single surgeon from January 2017 through March 2022 were retrospectively analysed. All patients received either original or altered formulated nutritional supplementation throughout the study. In total, three formulae, slightly different from each other, were used at three different time points to formulate the supplement.

**Results:**

In total, 196 patients were included. The average age and preoperative body mass index were 44.9 ± 6.7 years and 43.6 ± 22.5 kg/m^2^, respectively. Nutritional follow-up was available on 77.5%, 73.2%, 73.4%, and 59.7% of patients at 12, 24, 36, and 48 months, respectively. At baseline, 48.3%, 30%, 14.9%, 13.3%, 12.4%, 3.8%, 2.3%, and 0.5% of the patients had vitamin D, calcium, folic acid, total protein, iron, vitamin B12, copper, and vitamin A deficiencies, respectively. Postoperatively, mild to moderate vitamin deficiencies were noted in 14.2% of the patients in the first 18 months; however, at 4 years, the cohort had zero nutritional deficiencies. There were no long-term complications, revisions/conversions, or mortalities related to nutritional deficiencies.

**Conclusion:**

Factors, like preoperative and postoperative early, aggressive correction of nutritional deficiencies, regular laboratory monitoring and follow-ups with the multidisciplinary team, and adherence to our formulated nutritional supplement, have contributed to favourable nutritional outcomes at 4 years.

**Graphical Abstract:**

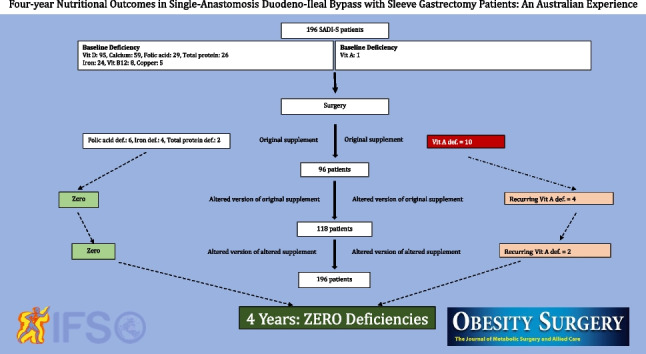

The single-anastomosis duodeno-ileal bypass with sleeve gastrectomy (SADI-S) surgery was introduced to minimise the risk of complications such as nutritional deficiencies, which are associated with traditional duodenal switch (DS) [1, 2].

Unlike the traditional DS surgery, SADI-S has been performed using a loop, 200-, 250-, and 300-cm common channel lengths. The long-term outcomes of this surgery using different limb lengths are already established in the literature [1–3]. The overall outcomes with SADI-S (300 cm) are favourable, with an acceptable rate of nutritional deficiencies compared to other malabsorptive/combination surgeries [2, 4–8]. Despite this, nutritional deficiency is still a major concern.

The extent of malnutrition, as well as the overall outcomes of SADI-S or other malabsorptive surgery, is primarily dependent on the length of the common channel. The ideal length of the common channel in SADI-S to minimise malnutritional deficiencies is currently unknown. It is currently believed that the risk of malnutritional deficiencies associated with this procedure can be reduced with regular clinic follow-ups with a multidisciplinary team. The major factors associated with decreasing the risk of minimising the potential risk of malnutrition following such procedures include adequate nutritional supplementation, regular recommended blood testing, close monitoring for signs and symptoms of nutritional deficiency, and the costs of supplementation [9–11].

There are some commercial nutritional supplements available on the market for patients who have undergone bariatric surgery; however, there is no nutritional supplement specifically formulated for SADI-S patients. There is a particular lack of access to specifically designed bariatric nutritional supplements in Australia, which is a potential barrier to increasing the use of the SADI-S procedure despite its favourable outcomes.

This article aimed to present the clinical-based, mid-term outcomes of a nutritional supplement designed for SADI-S patients using the American Society for Metabolic & Bariatric Surgery (ASMBS) guidelines [11].

## Methods

A formal non-research determination was sought from our local institution to perform the SADI-S surgery, which was approved by the medical advisory committee. This is a retrospective analysis of data from 196 consecutive patients who had undergone a primary SADI-S procedure performed by one surgeon at a single private institute from January 2017 through March 2022. Prior to undergoing surgery, all eligible patients signed a consent form which included consent to use de-identified data for scientific and research purposes. The risk and benefits of different bariatric procedures were explained to them in detail. Patients were asked to follow up after surgery at 2 weeks, 3 months, 6 months, 9 months, 12 months, 18 months, 24 months, and then every year. All patients were evaluated using a multidisciplinary team approach. Patients were educated before and after surgery on the expected nutrient deficiencies that are associated with the procedure as a result of alterations in physiology.

Nutritional deficiencies were aggressively corrected prior to surgery. The majority of the cohort was sent to the same laboratory for pre- and postoperative blood work to minimise the risk of bias. Vitamin deficiencies were defined in accordance with the ASMBS 2016 guidelines [11]. Mid-term outcome was defined as outcomes more than 3 years and less than 5 years.

When we started performing SADI-S, Australia did not have commercially available bariatric nutritional supplements that cater to any specific bariatric surgery [5]. This issue was discussed with the Therapeutic Goods Australia (TGA). Per their advice, our practice used a clinical compounding pharmacy to compound and dispense our own vitamin and mineral supplements to our SADI-S patients. The original supplement used in the study and its altered versions were mainly formulated using the ASMBS nutritional 2016 updated guidelines [11].

A dedicated compounding laboratory and trained personnel were used to compound the supplements. A certificate of analysis was also obtained. All ingredients used in compounding the supplement were sourced from TGA-approved suppliers and were thoroughly checked to ensure correct dosing and form. Three slightly different formulae were used to formulate the supplements at three different time points during the study period. The only alteration in the formulae was the change from vitamin A to beta-carotene and its dosages. All patients in the present study received either original or altered and formulated nutritional supplementations throughout the study duration.

The original nutritional supplement contained 300 0IU of vitamin A as an ingredient and was prescribed to the first 96 (48.9%) patients from postoperative months 6 through 31 (duration: 25 months) (Table 1). From months 32 through 36 (duration: 5 months), an altered version of the original supplement containing 1460 IU of beta-carotene in place of the vitamin A was prescribed to 118 (60.2%) patients (Table 1). The original 96 participants were also transitioned onto the modified supplements. From month 37 until the conclusion of the study (month 48 [duration: 12 months]), an altered version of the altered supplement was prescribed to 196 patients which contained 5000 IU of beta-carotene (Table 1). In this version, the dosage of beta-carotene was increased from 1460 to 5000 IU.

**Table 1:** Nutritional supplements and time frames

Ingredient	June 2017*	August 2019	Current
Vitamin B1 (thiamine)	12 mg	12 mg	12 mg
Vitamin B12 (methylcobalamin)	500 μg	500 μg	500 μg
5-MTHF (folate)	800 μg	800 μg	800 μg
Vitamin A (acetate)	3000 IU	Nil	Nil
Beta-carotene	nil	1460 IU	5000 IU
Vitamin E (succinate)	20 IU	20 IU	20 IU
Vitamin K1	300 IU	300 IU	1 mg
Vitamin D3	3000 IU	3000 IU	3000 IU
Iron (picolinate)	18 mg	18 mg	18 mg
Zinc (picolinate)	18 mg	18 mg	18 mg
Copper gluconate	2 mg	2 mg	2 mg
Pyridoxine HCL	nil	5 mg	5 mg

*Formulated using the ASMBS nutritional guidelines 2016 update

Abbreviation: *mg* milligram, *μg* microgram, *IU* international unit

The nutritional data were automated using “DataMedic©” (Patient and Cohort Analysis software system). The software system communicated with pathology laboratories using FHIR® Standard (HL7R®). “DataMedic” parsed electronic messages and matched the patient’s name and date of birth with existing patient details in order to store pathology results, as well as the date and time of the test in the patient database.

To compare the nutritional markers before and after surgery, we employed a non-parametric Mann-Whitney test and computed *U* statistic [12, 13]. ${U}_1={R}_1-\frac{n_1\left({n}_1+1\right)}{2}$, where *R*_1_ is the sum of the ranks for group 1 and *n*_1_ is the number of readings in group 1. ${U}_2={R}_2-\frac{n_2\left({n}_2+1\right)}{2}$, where *R*_2_ is the sum of the ranks for group 1 and *n*_2_ is the number of readings in group 2.

Following surgery, patients were categorized into seven groups (3, 6, 12, 18, 24, 36, and 48 months), based on months elapsed since surgery. The pre-surgery cohort readings for nutritional markers were grouped and labelled as group 0 to compare with group 3 (3 months), group 6 (6 months), group 12 (12 months), group 18 (18 months), group 24 (24 months), group 36 (36 months), and group 48 (48 months).

The NULL Hypothesis (H_0_) is that nutritional marker readings do not differ significantly pre-surgically (group 0) when compared to post-surgical readings. Results are reported at a 95% confidence interval and sufficient statistical significance at a *p*-value of ≤0.05.

### Operative Technique

Our surgical technique for SADI-S has been described previously [5]. In all SADI-S cases, a common channel of 300 cm was performed.

The major steps involved in performing a laparoscopic SADI-S procedure include walking of the small bowel, tacking of the small bowel, dissection of the greater curvature of the stomach, duodenal dissection, sleeve creation, duodenal transection, creation of the duodeno-ileal (DI), and the leak test [5]. The method used to perform laparoscopic SADI-S surgery for the cohort in this study is detailed below.

The first step was to locate the ileocecal valve. The small bowel was counted 300 cm proximal to the ileocecal valve. The antimesenteric border of the bowel at this point was attached loosely to the omentum just below the pyloric valve to mark the site for anastomosis. This suture was later cut prior to the duodeno-ileostomy being performed.

The greater omentum of the stomach was resected using a similar technique used in sleeve gastrectomy (SG). The dissection was then carried medially to the second and third portions of the duodenum. Retrogastric and retroduodenal adhesions were taken down with the LigaSure device. The limit of this dissection was determined by the gastroduodenal artery embedded in the pancreas. This way, we had at least 3 to 4 cm of duodenum dissected beyond the pylorus. It is our preference not to take down the right gastric artery routinely, as in almost all cases, there was enough space created by this dissection method to introduce the stapler safely without any blunt dissection. Once we were sure that the duodenal transection would be possible safely, we then moved on to do the SG portion of the procedure, using a 36-French bougie for sizing. The gastric resection began 4 to 6 cm away from the pylorus, hugging loose on the bougie and ending 1 to 2 cm off the angle of His. The first two stapler firings were with 45-mm black Endo GIA™ reinforced Tri-Staples™. The next firings were with 60-mm Black Endo GIA™ reinforced Tri-Staples™.

The duodenum was then transected at least 2–3 cm beyond the pylorus with 60-mm purple Endo GIA™ reinforced Tri-Staples™. We then created an end-to-side (duodenum-to-ileum) anastomosis. The enterotomy was closed with a running posterior layer and a running anterior layer using 2 “0” Polysorb sutures with the Endo Stitch Device (Medtronic).

## Results

Overall, 196 suitable patients were identified for analysis. The study cohort consisted of 30.6% males and 69.4% females, with an average age of 44.9 ± 6.7 years. The average preoperative body mass index (BMI) was 43.6 ± 22.5 kg/m^2^.

At the time of data analysis, 196, 176, 128, and 97 patients were out 12, 24, 36, and 48 months, respectively. The nutritional follow-up was available on 152 patients (77.5%), 129 patients (73.2%), 94 patients (73.4%), and 58 patients (59.7%) at 12, 24, 36, and 48 months, respectively.

**Fig. 1:**
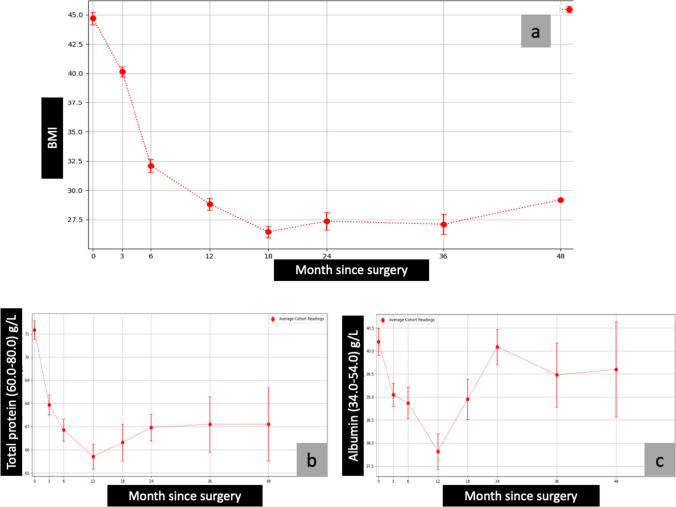
BMI, TP, and albumin vs. month since surgery. Abbreviation: BMI, body mass index; 0= baseline; TP, total protein

The average nadir weight was noted to occur at approximately 18 months after surgery and remained stable over the next 18 months (Fig. 1a). Slight weight regain was noted after 36 months had elapsed since the surgery. At 4 years, the average BMI of the study cohort was close to 29 kg/m^2^.

### Nutritional Outcomes

#### Average

##### Preoperative and Postoperative

**Fig. 2:**
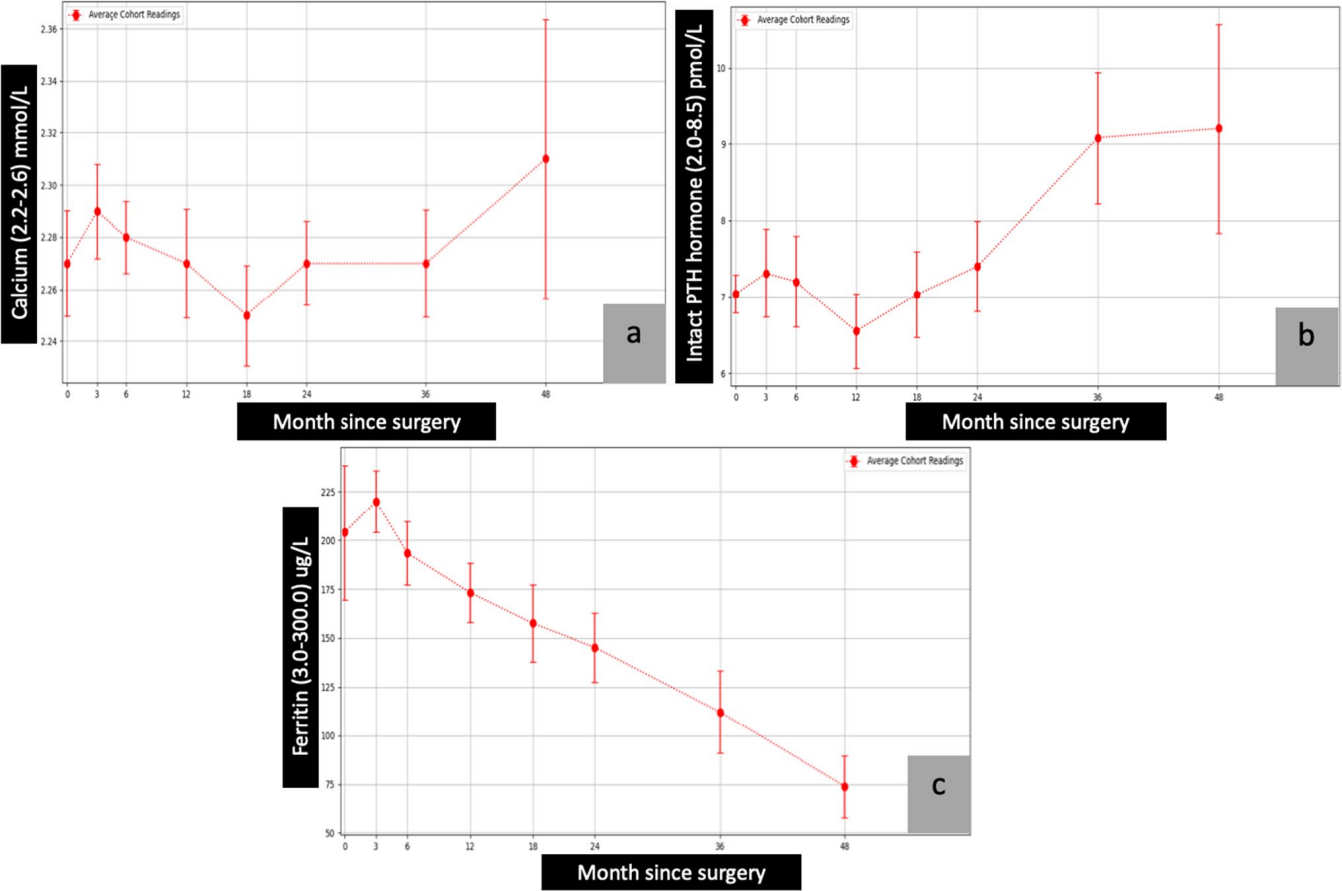
Calcium, PTH, and ferritin vs. month since surgery. Abbreviation: 0= baseline; PTH, parathyroid hormone

**Fig. 3:**
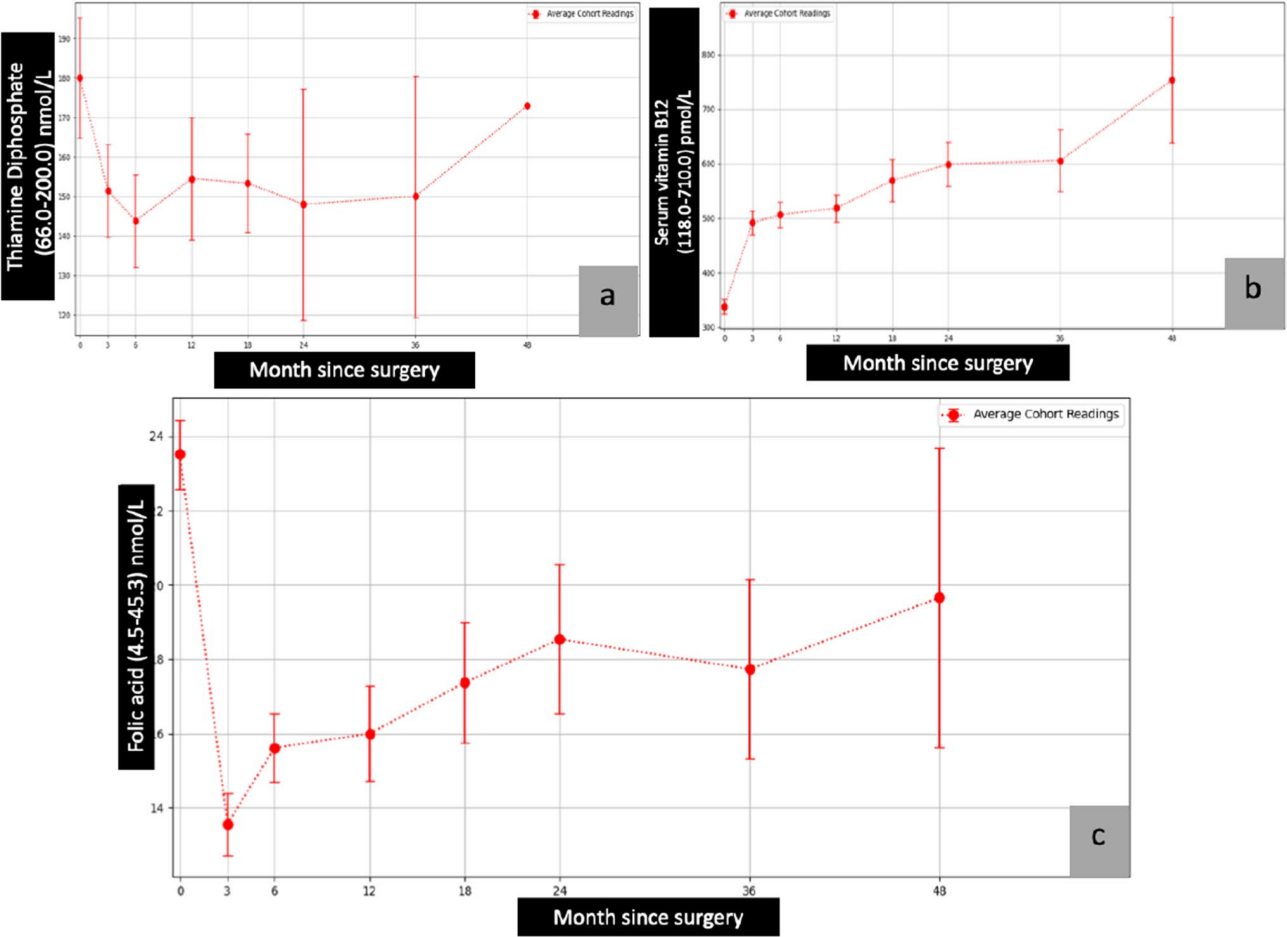
Vitamin B1, vitamin B12, and folic acid vs. month since surgery. Abbreviation: 0=baseline

**Fig. 4:**
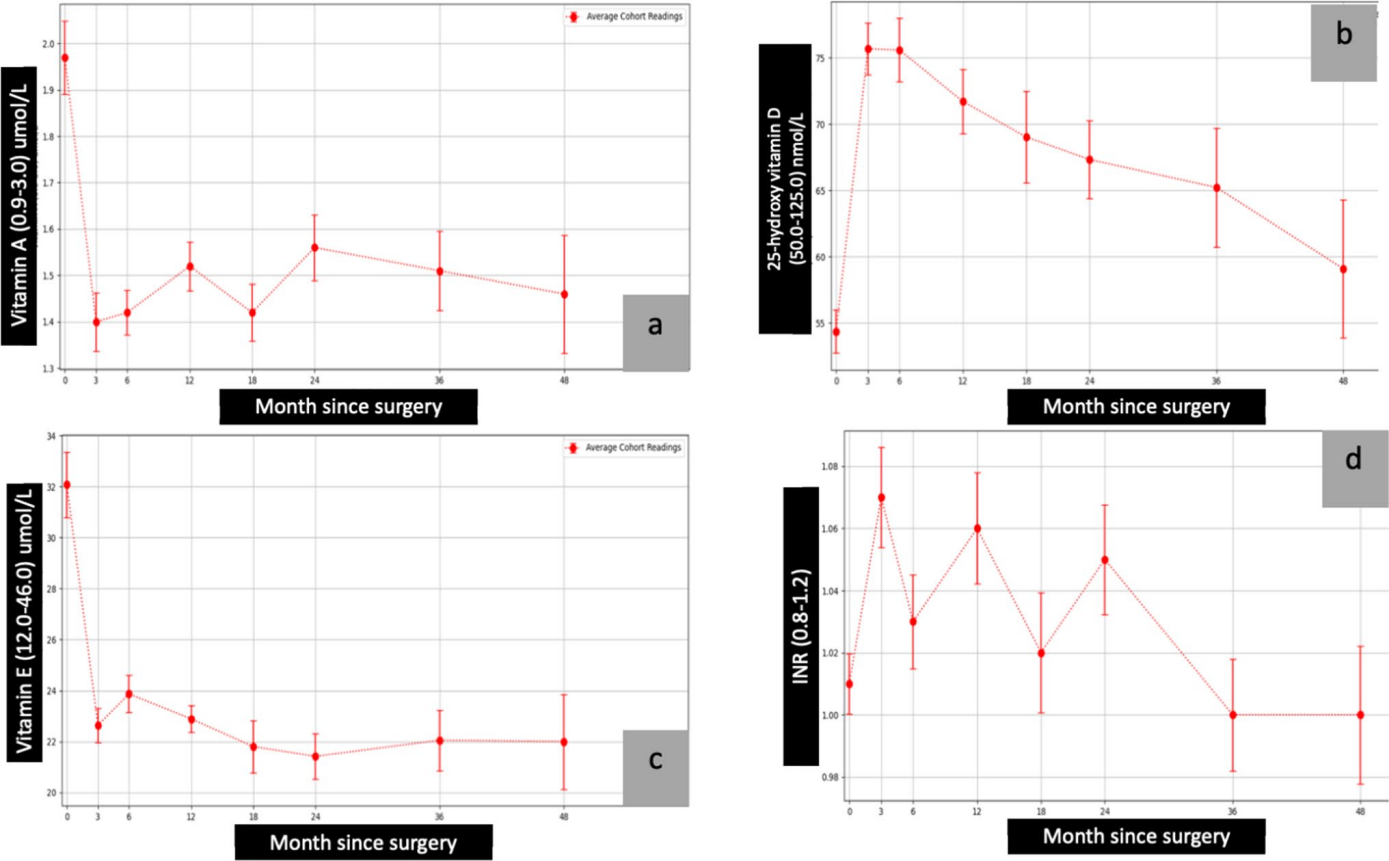
Vitamin A, vitamin D, vitamin E, and INR vs. month since surgery. Abbreviation: 0= baseline; INR, international normalised ratio

**Fig. 5:**
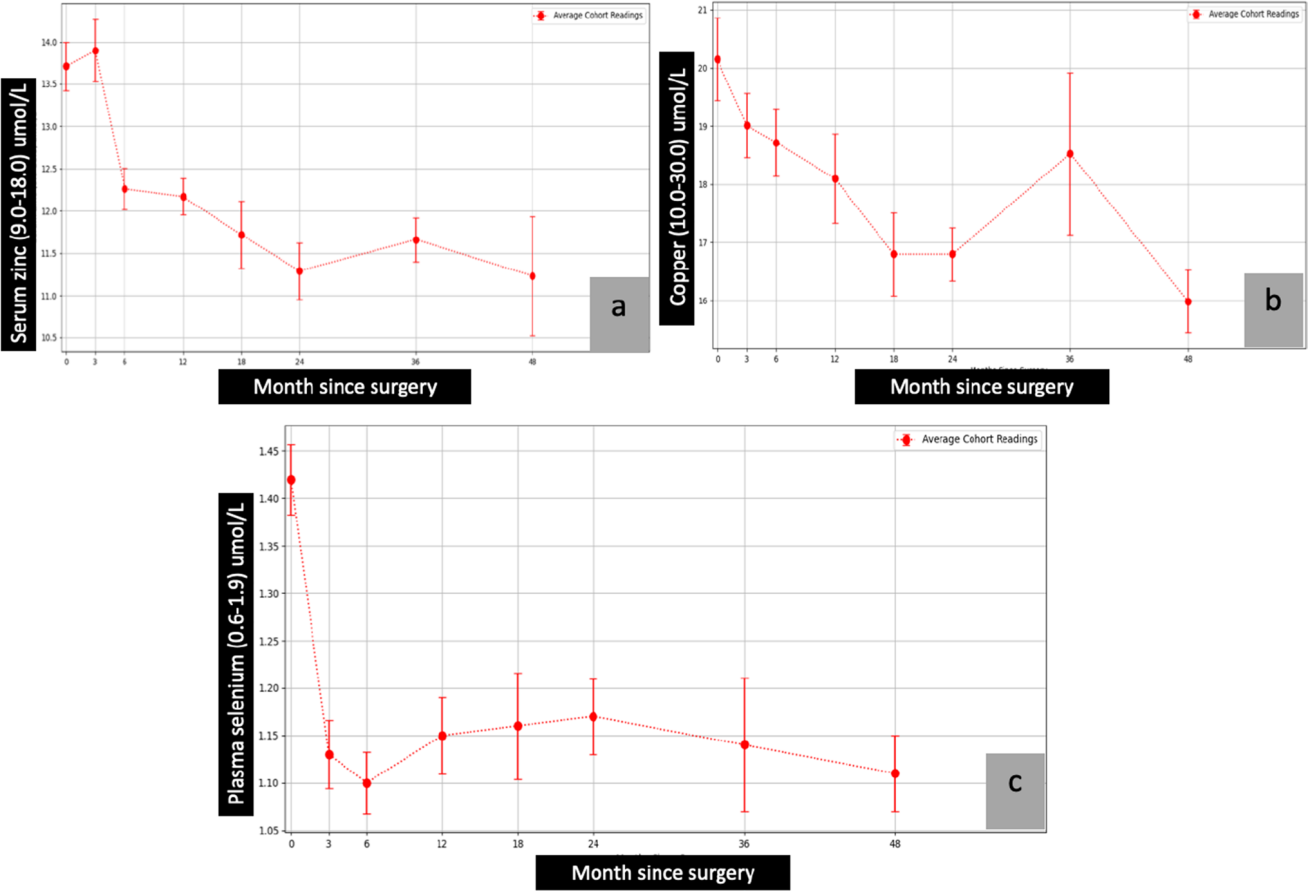
Zinc, copper, and selenium vs. month since surgery. Abbreviation: 0=baseline

The average of pre- and post-surgical total protein, albumin, calcium, parathyroid hormone (PTH), ferritin, vitamin B1, vitamin B12, folic acid, vitamin A, vitamin D, vitamin E, international normalised ratio (INR), zinc, copper, and selenium are presented in graphical forms (Figs. 1, 2, 3, 4, and 5)

##### Trend

The average total protein and albumin levels dropped significantly at approximately 12 months post-procedure but remained within the accepted normal range (Fig. 1b and c). Improvement in total protein and albumin levels was noted from 13 months through 48 months. A similar pattern was noted in the levels of vitamins A and E (Fig. 4a and c).

The present study had a 69.4% female population. The average ferritin levels gradually dropped over months from the time of surgery; however, they were still in the normal range throughout the study period (Fig. 2c).

The INR showed a zig-zagging rise and fall (range 1.1–1.7) through the first 24 months of the study; INR readings stabilised in all participants from month 24 to month 48 (Fig. 4d).

The average levels of vitamin D remained stable in the first few months following surgery, with a gradual decline in levels noted throughout the 48 months of the study (Fig. 4b). Despite the gradual drop, the average levels remained in the normal range. Average levels of calcium began to rise after 36 months, leading to a plateau in serum PTH levels (Fig. 2a and b). The average levels of PTH were stable from 36 to 48 months. A similar trend was noticed in vitamins B1 and B12 and folic acid, with average levels elevated after 36 months (Fig. 3a, b, and c). The average levels of zinc, copper, and selenium gradually decreased over the first 24 months (Fig. 5a, b, and c). However, the levels stabilised and stayed in the normal range from 24 to 48 months.

#### Prevalence and Incidence

##### Preoperative

At baseline, 48.3% of the patients had vitamin D deficiency, 30% of the patients had calcium deficiency, 14.9% of the patients had a folic acid deficiency, 13.3% of the patients had protein deficiency, 12.4% of the patients had iron deficiency, 3.8% of the patients had vitamin B12 deficiency, 2.3% of the patients had a copper deficiency, and 0.5% patient had vitamin A deficiency.

##### Postoperative Outcome

In the first 18 months after surgery, overall, 14.2% of the patients experienced mild or moderate deficiency of any kind.

**Fig. 6:**
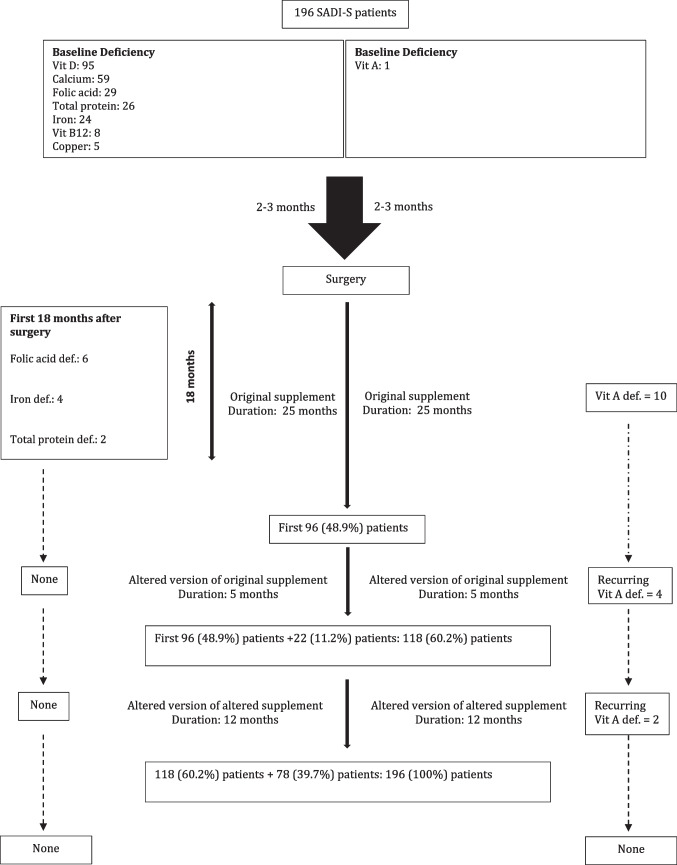
Flow chart detailing the change in regimens and the outcomes of the supplement

In total, 16 (8.1%) patients (13 female [81.2%] and 3 males [18.7%]) experienced mild to moderate vitamin A deficiencies (Fig. 6). Of those 16 patients, 10 (62.5%) patients were deficient from the original supplement, which had 3000 IU of vitamin A. Of those 10 patients, eight (80%) patients had mild, and two (20%) patients had moderate vitamin A deficiencies. Of those 10 patients, five patients presented with deficiencies between 3 and 6 months postoperatively. Four patients presented with deficiencies between 12 and 19 months postoperatively.

## Vitamin A

Four (25%) patients (4/16 vitamin A deficiency patients) were deficient from the altered version of the original supplement, which had 1460 IU beta-carotene. Three patients had mild and one patient had moderate deficiencies. Of those four patients, three patients had deficiencies between 3 and 12 months and were treated with oral supplements. One patient with moderate deficiency had reoccurring events at 12, 18, and 39 months. This patient received oral supplementation at 12 months, intravenous (IV) infusion at 18 months, and oral supplementation at 29 months.

Two (12.5%) patients (2/16 vitamin A deficiency) were deficient from the altered version of the altered supplement at 6 months. Both patients had one course of oral supplementation with a positive outcome.

## Other Nutritional Deficiency

Six (3%) patients experienced mild folic acid deficiency, with levels ranging between 3 and 4.9 nmol/L. Two (1%) patients experienced mild protein (33 g/L), and four (2%) patients experienced iron deficiencies. Most of the deficiencies were diagnosed between 3 and 9 months postoperatively. They were aggressively treated; however, despite the correction, recurrent vitamin A deficiency was noted until 18 months.

At 48 months, none of the patients in the cohort had abnormal levels of vitamin D, calcium, folic acid, total protein, iron, vitamin B12, copper, or vitamin A that were seen preoperatively.

### Complication and Conversion

The rate of nutritional-related long-term complications and rate of revision/conversion to another bariatric surgery was 0%.

## Discussion

The 4-year nutritional outcomes in SADI-S patients using our formulated nutritional supplement are favourable. Despite 14% of patients experiencing a mild to moderate deficiency in the first 18 months post-surgery, none of the patients had recurring or new nutritional deficiency at 4 years. In our opinion, one of the major contributors to the favourable outcomes of the formulated nutritional supplement was the 2016 update to the ASMBS guidelines, which provided expert-based and evidence-based supplementation guidelines [11]. In the present study, the original guidelines were used as a starting point and were slightly modified to formulate the final version of the supplement.

One of the major challenges facing the use of SADI-S surgery in Australia is the lack of commercially available bariatric nutritional supplements specifically designed for post-SADI-S patients. Australia also lacks commercially available bariatric nutritional supplements which cater to any specific bariatric procedure, meaning that patients and doctors are challenged with developing an appropriate regime of multiple supplements. In recent years, SADI-S has gained popularity in the bariatric world. With proper nutritional supplements, the potential risk of malnutrition with SADI-S can be minimised [2, 11]. Combination surgeries like DS, SADI-S, and Roux-en-Y gastric bypass (RYGB) require more daily nutritional supplementation to maintain normal levels of essential vitamins and minerals when compared to traditional surgeries [14].

The second challenge in adequate nutritional supplementation following SADI-S procedures is the cost of life-long supplementation. Malabsorptive/combination surgeries require patients to adhere to a regime of life-long nutritional supplementation; however, the long-term adherence to nutritional supplementation following bariatric surgery is often poor [15]. One of the major factors that influence patient adherence to nutritional supplements is the cost [16]. Supplements must be affordable and cost-effective for patients to continue taking them for a lifetime. In the present study, per our suggestion, the first few patients opted for commercial bariatric supplements that are easily available in the USA. The original monthly supply cost in the USA is around 40 US dollars; however, after shipping internationally, they cost around 120 Australian dollars. It was not feasible for our patients to continue taking them, and that is why our practice approached TGA to discuss this issue in detail. Our formulated nutritional supplement is cost-effective for patients in Australia. The monthly supply costs 29.95 Australian dollars, which includes iron, calcium, and all the multivitamins involved in the formulation. The formulation used in the study was 75% cheaper for patients than the commercial alternative, and in the opinion of the authors, this cost reduction is likely to have contributed to the favourable outcomes of the study.

The third challenge noted during the study period was recurrent vitamin A deficiency in the first 18 months following the procedure. SADI-S is a modification of traditional DS; hence, our original compounded supplement was formulated using the ASMBS nutritional guidelines for traditional DS [11]. Typically, the length of the common channel in traditional DS is around 100 cm [17]. We postulated that since the length of the common channel in our SADI-S cases is 300 cm, we would require 1/3 of the recommended dosage by the ASMBS for traditional DS (10000 IU/day), which will be around 3000 IU/day. Our assumption of 3000 IU/ day based on the common channel length was refuted by the early results of the study. With the original version of the supplement, which contained 3000 IU/day of vitamin A, our cohort had 10 (10.4%) vitamin A deficiencies in the first few years. Only in those cases, the dosage was increased to 5000 IU/per day. The current literature suggests that in pregnant women who are vitamin A deficient, supplementation at doses lower than 10,000 IU/day or 25,000 IU/week is beneficial and does not have an associated risk of teratogenicity to the foetus [18].

The fourth challenge was the high dosage of vitamin A in women of reproductive age. The average age of menopause in Australian women is 51 years (range, 45–55 years) [19]. Our cohort had a 69.4% female population, with an average age of 46.5 ± 9.9 years. After a thorough discussion with our multidisciplinary team and a literature review, a decision was made to replace the vitamin A in the original supplement with a precursor, beta-carotene, which is considered relatively safer than vitamin A itself, especially in pregnancy.

The altered version of the supplement contained beta-carotene at a dosage of 1460 IU/day. At this dosage, there were still four vitamin A deficiencies. It is likely that an error was made in calculating the conversion rate of beta-carotene to vitamin A, which may have resulted in some cases of avoidable vitamin A deficiency. After rectifying the conversion error, the dosage was increased to 5000 IU/day with a positive outcome. At a dose of 5000 IU/day of beta-carotene, only two patients had a vitamin A deficiency at 6 months which required a course of oral supplementation. The option of increasing the dose of beta-carotene to 10,000 IU/day was considered but ultimately rejected as being economically unwise due to the associated total cost increase of the supplement regime in comparison with the small number of patients with persistent vitamin A deficiencies. Following these changes, at 48 months, our cohort had only 0% vitamin A deficiencies.

Maximal weight loss was seen in the first 18 months of the study period. The average BMI at 48 months was 29 kg/m^2^, which is in keeping with existing evidence. Surve et al., in their long-term outcome article, reported an average BMI of 30.1 kg/m^2^ at 48 months [2]. Sánchez-Pernaute et al. reported an average BMI of 27.5 kg/m^2^; however, a majority of patients in their cohort received a 250-cm (60%) or 200-cm (30%) common channel [6]. Only 9% of the cohort received a 300-cm common channel. In their cohort, at 60 months, low levels of total protein, vitamin D, ferritin, vitamin A, and zinc were noted in 25%, 70%, 56%, 40%, and 32% of the study population, respectively. With a common channel of 300 cm, Surve et al. reported that compared with baseline, calcium, PTH, albumin, total protein, and vitamin E worsened significantly at 60 months [2]. A recent long-term outcome study by Ortiz-Zuñiga et al. compared the outcomes of SADI-S with <250 cm and >250 cm [20]. They found that a common channel of <250 cm was associated with severe malabsorptive complications that required surgical correction. In the present study, with a common channel length of 300 cm, the nutrient stores of most nutrients were depleted within the first 18 months, remained stable from 24 to 36 months, and continued an upward trend until the end of the study. This correlated with the physiology of the surgery. If true, it may still trend upwards after the actual “intestinal adaption” after 60 months.

Supplementation non-adherence can have negative consequences, especially with any malabsorptive procedure. To avoid information and misclassification bias, adherence to drug regimens is generally monitored using the body fluid concentrations of the substance in question. However, the measurement of serum concentrations is time-consuming and inconvenient. As such, drug adherence is often monitored using self-report or clinician assessment during follow-up appointments. In the present study, the postoperative supplementation adherence rate was 100%. The high adherence rate is likely the result of preoperative and continuing postoperative education regarding the importance of nutritional supplementation following the SADI-S surgery. All postoperative follow-up appointments were in person. Clinic visits were coordinated with a bariatric team, where drug adherence and compliance-related questions were asked regardless of health-related complaints. In addition, at each follow-up visit, patients were also evaluated for clinical signs and symptoms related to nutritional deficiencies. The clinical compounding pharmacy used script refill reminders to remind patients when medication refills were due. Patients also received reminders of when they were due to refill their medication and when they were due for repeat blood testing through the compounding pharmacy and the pathology service.

The findings of this study should be considered in light of several limitations. The main limitation of the study was the use of three different formulations of nutritional supplementation at different time points throughout the study. The nutritional outcomes at 48 months are the overall results of all three formulae, along with other influencing factors. However, the first 96 (48.9%) patients subsequently received all three versions of the supplement, 22 (11.2%) patients did not receive the original supplement (3000 IU vitamin A), and the last 79 (40.3%) patients in the cohort did not receive the first two versions (3000 IU vitamin A/1460 IU beta-carotene) of the supplement. The only change in all three formulae was the dosage and form of vitamin A to avoid teratogenicity in pregnancy. The second limitation of the study was the duration of each version of the supplement. The altered version of the original supplement was prescribed for only 5 months. The ASMBS guidelines recommend postoperative blood testing every 3 months for the first year, every 6 months for the second year, and annually thereafter, regardless of the type of bariatric procedure performed [21]. One hundred and eighteen patients received the altered version of the supplement for a duration of 5 months. Of those 118 months, the majority of the patients were 12 months post-surgery before starting the altered formula and may not have received their annual blood work within the 5-month duration of the second formulation. This means that the true nutritional outcomes associated with this formulation were likely not fully evaluated in the study period. In total, four (3.3%) patients had vitamin A deficiencies from the altered version of the original supplement. As a result, this estimate of vitamin A deficiency on the first altered formula is likely to be inaccurate. The third limitation of this study was the percentage follow-up. Typically, in bariatrics, nutritional follow-up rates are lower than the regular clinic follow-up rates. In the present study, at 48 months, 97 patients were out; however, our nutritional follow-up at 48 months was close to 60% despite Australia’s strict COVID-19 travel restrictions. The retrospective nature of the study was the fourth limitation. The fifth limitation was monitoring INR/PT instead of vitamin K. In our opinion, INR and PT are more reliable than vitamin K. There is no available data on vitamin K deficiencies in pre-weight loss surgery patients. In the present study, vitamin K was measured in a few patients. The cost of testing vitamin K levels was high in comparison to its clinical benefit, as all tests are needed to be sent to a different city (Sydney). Moreover, the measurement of vitamin K does not relate to clinical symptoms [22]. The monitoring of vitamin K levels is usually through prothrombin time (PT) and INR. These values measure the presence of vitamin K-dependent factors, which is especially important in patients with warfarin toxicity or vitamin K-related coagulopathies. Hence, we changed to measure INR/PT.

Despite these limitations, the study also had several strengths. The use of software to automate nutritional data entry directly from pathology laboratory results to patient records saved time and minimised the potential for systemic bias due to information bias resulting from human error. The supplement was formulated using the ASMBS 2016 updated guidelines for traditional DS surgery, and TGA-approved ingredients, sourced from TGA-approved suppliers only. This is the only article in the literature that focused on the outcomes of a nutritional supplement solely formulated for SADI-S patients. Moreover, the overall results of the supplement are promising. The final version of the supplement used in this study can readily be implemented by practices in locations that do not have readily available bariatric nutritional supplements and where compounding supplements would lower the cost of ongoing supplementation, improving outcomes and adherence.

## Conclusions

The results of our study show promising results, with favourable nutritional outcomes achieved in patients given a targeted nutritional supplement following SADI-S procedures. This supplementation regime can be easily employed to improve postoperative outcomes in areas that struggle with access to commercially available bariatric nutritional supplementation or in which cost limits adherence to postoperative supplementation adherence. Additional factors contributing to positive postoperative outcomes in this study include early and aggressive correction of nutritional deficiencies pre- and postoperatively, regular laboratory testing, and multidisciplinary team follow-up. These results also show the need for ongoing research into the appropriate dosing of vitamin A and other nutrients following newer malabsorptive and combination-type weight-loss surgeries.

## References

[1] Sánchez-Pernaute A, Rubio MÁ, Cabrerizo L, Ramos-Levi A, Pérez-Aguirre E, Torres A (2015). Single-anastomosis duodenoileal bypass with sleeve gastrectomy (SADI-S) for obese diabetic patients. Surg Obes Relat Dis.

[2] Surve A, Cottam D, Medlin W, Richards C, Belnap L, Horsley B, Cottam S, Cottam A (2020). Long-term outcomes of primary single-anastomosis duodeno-ileal bypass with sleeve gastrectomy (SADI-S). Surg Obes Relat Dis.

[3] Topart P, Becouarn G (2017). The single anastomosis duodenal switch modifications: a review of the current literature on outcomes. Surg Obes Relat Dis.

[4] Yashkov Y, Bordan N, Torres A, Malykhina A, Bekuzarov D (2021). SADI-S 250 vs Roux-en-Y duodenal switch (RY-DS): results of 5-year observational study. Obes Surg.

[5] Surve A, Rao R, Cottam D, Rao A, Ide L, Cottam S, Horsley B (2020). Early outcomes of primary SADI-S: an Australian experience. Obes Surg.

[6] Sánchez-Pernaute A, Herrera MÁR, Ferré NP, Rodríguez CS, Marcuello C, Pañella C, Antoñanzas LL, Torres A, Pérez-Aguirre E (2022). Long-term results of single-anastomosis duodeno-ileal bypass with sleeve gastrectomy (SADI-S). Obes Surg.

[7] Surve A, Cottam D, Richards C, Medlin W, Belnap L (2021). A matched cohort comparison of long-term outcomes of Roux-en-Y gastric bypass (RYGB) versus single-anastomosis duodeno-ileostomy with sleeve gastrectomy (SADI-S). Obes Surg.

[8] Surve A, Cottam D, Belnap L, Richards C, Medlin W (2021). Long-term (> 6 years) outcomes of duodenal switch (DS) versus single-anastomosis duodeno-ileostomy with sleeve gastrectomy (SADI-S): a matched cohort study. Obes Surg.

[9] Lupoli R, Lembo E, Saldalamacchia G, Avola CK, Angrisani L, Capaldo B (2017). Bariatric surgery and long-term nutritional issues. World J Diabetes.

[10] Sherf Dagan S, Goldenshluger A, Globus I, Schweiger C, Kessler Y, Kowen Sandbank G, Ben-Porat T, Sinai T (2017). Nutritional recommendations for adult bariatric surgery patients: clinical practice. Adv Nutr.

[11] Parrott J, Frank L, Rabena R, Craggs-Dino L, Isom KA, Greiman L (2017). American Society for Metabolic and Bariatric Surgery integrated health nutritional guidelines for the surgical weight loss patient 2016 update: micronutrients. Surg Obes Relat Dis.

[12] Bucchianico D (1999). Combinatorics, computer algebra, and the Wilcoxon-Mann-Whitney test. J Stat Plan Infer.

[13] Fay MP, Proschan MA (2010). Wilcoxon-Mann-Whitney or t-test? On assumptions for hypothesis tests and multiple interpretations of decision rules. Stat Surv.

[14] Lange J, Königsrainer A (2019). Malnutrition as a complication of bariatric surgery - a clear and present danger?. Visc Med.

[15] Brorsson AL, Nordin K, Ekbom K (2020). Adherence to vitamin supplementation recommendations in youth who have undergone bariatric surgery as teenagers: a mixed methods study. Obes Surg.

[16] Kardas P, Lewek P, Matyjaszczyk M (2013). Determinants of patient adherence: a review of systematic reviews. Front Pharmacol.

[17] Anthone GJ, Lord RV, DeMeester TR, Crookes PF (2003). The duodenal switch operation for the treatment of morbid obesity. Ann Surg.

[18] Dibley MJ, Jeacocke D (2001). Safety and toxicity of vitamin A supplements in pregnancy. Food Nutr Bull.

[19] Jones EK, Jurgenson JR, Katzenellenbogen JM, Thompson SC (2012). Menopause and the influence of culture: another gap for Indigenous Australian women?. BMC Womens Health.

[20] Ortiz-Zuñiga AM, Costa Forner P. Cirera de Tudela A, Garcia Ruiz A, et al. The impact of the length of the common intestinal loop on metabolic and nutritional outcomes of patients with severe obesity who undergo of single anastomosis duodeno-ileal bypass with sleeve gastrectomy: 5-year follow-up. J Laparoendosc Adv Surg Tech A. 2022;1 10.1089/lap.2021.0863. Epub ahead of print 10.1089/lap.2021.0863 35363561

[21] Menser T, Muniz Castro J, Lopez A, Jones SL, Kash BA, Sherman V, Tariq N (2020). Post-bariatric surgery lab tests: are they excessive and redundant?. Surg Endosc.

[22] Imbrescia K, Moszczynski Z (2022). Vitamin K. StatPearls.

